# Physical Activity Profiles among Patients Admitted with Acute Exacerbations of Chronic Obstructive Pulmonary Disease

**DOI:** 10.3390/jcm12154914

**Published:** 2023-07-26

**Authors:** Christopher Byron, Christian R. Osadnik

**Affiliations:** 1 Department of Physiotherapy, Monash University, Melbourne 3199, Australia; chris.byron@monash.edu; 2 Monash Lung Sleep Allergy Immunology, Monash Health, Melbourne 3168, Australia

**Keywords:** physical activity, inpatient, hospital admission, disease exacerbation, COPD

## Abstract

People with hospitalised acute exacerbations of chronic obstructive pulmonary disease (AECOPD) exhibit low levels of physical activity (PA) and increased risks of future exacerbations. While methods to objectively measure and express PA are established for people with stable COPD, less clarity exists for people during AECOPD. Further, the relationship between PA during AECOPD and clinically relevant outcomes remains uncertain. The purpose of the study was to evaluate how much PA (step count and intensity) people accumulate during hospitalised AECOPDs, and the effect of accumulated inpatient PA (expressed via differing metrics) on length of stay (LOS), PA recovery, and readmission risk. This study was a secondary analysis of prospective observational cohort data collected with Actigraph wActiSleep-BT devices from patients with AECOPD in a Melbourne hospital from 2016 to 2018. Step counts and PA intensity throughout the hospital admission and at one-month follow-up were collected and analysed. Sixty-eight participants were recruited for inpatient measurement, and 51 were retained for follow-up. There were no significant changes in step count or intensity across the inpatient days, but 33/51 (65%) of participants demonstrated a clinically meaningful improvement in steps per day from 3817.0 to 6173.7 at follow-up. Participants spent most time sedentary and in light PA, with both PA metrics demonstrating significant influences on LOS and follow-up PA intensity, but with generally low explanatory power (R^2^ value range 7–22%). Those who had LOS < 5 days spent significantly less time sedentary and more time in light PA than those with LOS ≥ 5 days (*p* < 0.001 for both). Time spent sedentary or in light PA appears to be the most promising metric to associate with clinically relevant outcomes related to recovery following AECOPD. These findings can inform future clinical practice for the evaluation of inpatient PA to better establish its role in the clinical management of patients with AECOPD.

## 1. Introduction

Reductions in muscle mass, exercise tolerance [1], and physical activity (PA) levels are well established in people with chronic obstructive pulmonary disease (COPD) [2]. Low PA levels are associated with increased COPD-related primary care visits, hospital admissions, and all-cause mortality [3,4,5], and acute exacerbations of COPD (AECOPD) are associated with poor recovery of physical activity levels after discharge and a heightened risk for future readmission [6]. The precise determinants contributing to physical inactivity in this patient group are not fully understood, and little attention has been directed towards events that occur during the period of hospitalisation due to AECOPD.

The World Health Organisation recommends adults, including those living with chronic conditions, engage in at least 150 min of moderate-intensity PA and perform muscle-strengthening activities at least two days per week, and minimise periods of prolonged sitting [7]. This can be very challenging to achieve during times of hospitalisation, with prior systematic reviews suggesting that medical inpatients may spend as little as one hour per day in a standing or physically active state [8,9]. Among those experiencing an AECOPD, up to 87% of waking time may be spent sedentary [10], and step counts have been reported as low as 500–600 per day, on average [10,11]. Pitta et al. [12] observed that inpatients with AECOPD spent only 7% of their day in a weight-bearing position by day 2 of hospitalisation, which only increased to 9% by day 7 and to 19% one-month post-discharge. Many factors have been proposed to contribute to these observations, including constraints related to the hospital environment and patient beliefs [13,14]. Prior research suggests that approximately half of daily PA is accumulated by 13:00 h, and 75% by 18:00 h, with peaks between 09:00 and 12:00 and again at 14:00 [11]. This behaviour of inpatient PA profiles may reflect the nature of inpatient hospital environments, where medical, nursing, and allied health care and visiting hours may take place during these times. Physical activity levels do not appear to spontaneously resolve after discharge; however, the observed magnitudes of recovery among small patient samples from Brazil [12] and Australia [15] have varied. Various factors might contribute to this observation. For example, patients discharged from hospital with reduced lung function compared to premorbid levels have been shown to retain such lung function deficits for up to three months [16]. An important factor inhibiting our understanding of factors contributing to PA accumulation and recovery during AECOPDs is a lack of an agreed-upon method to undertake device-based assessments of PA levels during hospitalisation. International consensus for such evaluations exists for community-dwelling people with COPD (e.g., monitor use ≥8 h on ≥4 days/week) [17]; however, such methods are clearly impractical for hospital use where admission durations may, for example, be less than this recommended sampling frame.

An important technical evaluation of accelerometry-derived PA data from 389 hospital inpatients with AECOPD [11] suggested that a wear time of 11 h from any single inpatient day adequately captured PA levels. This sampling frame allowed for data to be recorded from peak morning times (8–11 a.m.) as well as afternoons (1–9 p.m.) and retained 80% of all relevant data. This insight is appealing in terms of the feasibility of objectively evaluating PA during AECOPD; however, it might overlook potentially relevant clinical information about individuals. For example, AECOPDs are heterogenous clinical events [18] and PA behaviours are likely to fluctuate throughout an admission. Individuals with mild respiratory symptoms might exhibit stable patterns of PA throughout their inpatient stay, while others might demonstrate progressive increases in PA as exacerbation symptoms resolve and one’s functional reserve improves [19]. Little is known of the potential relevance of such observations or fluctuations. Additionally, current knowledge is lacking regarding the most suitable metrics (e.g., step counts, time spent in light, moderate, vigorous, or moderate-to-vigorous PA (MVPA)) to express PA and their patterns of accumulation during AECOPD. This could be important as some metrics may offer greater sensitivity to change or associate more (or less) strongly with clinical outcomes than others. Data demonstrating the clinical relevance of various PA metrics during AECOPD are notably absent in the existing literature, thus prohibiting advancements in this field.

The present study, therefore, aimed to examine, among people with hospitalised AECOPDs, the patterns of PA accumulation across a range of relevant accelerometry metrics, and the clinical relevance of different approaches to express PA in order to clarify the potential impact upon future research and practice in this field.

## 2. Materials and Methods

A prospective observational cohort study was conducted and reported in accordance with STROBE guidelines [20]. The study was approved by the Human Research Ethics Committees of Monash Health (#HREC/16/MonH/367) and Monash University (#1614).

### 2.1. Study Procedures

Consenting participants were recruited from respiratory units of a major tertiary hospital in metropolitan Melbourne, Australia, between 2016 and 2018 if they had a clinical diagnosis (spirometrically confirmed or likely) of COPD, a smoking history of 10+ pack years, and were admitted to hospital due to AECOPD. Exclusion criteria comprised factors limiting participants’ ability to provide informed consent (e.g., written/spoke English language comprehension, severe cognitive deficits) or cooperate with test procedures (e.g., hemiparesis, severe osteoarthritis), and/or those with a poor prognosis (e.g., malignancy, palliation). A sequential convenience sample of 100 participants was planned as part of a broader exploratory cohort study from which data pertaining to this analysis were extracted. All participants received standard multidisciplinary care during their hospital admission, without the involvement of the researchers. Clinicians were not involved in data collection for this study.

### 2.2. Data Collection

**Figure 1:**
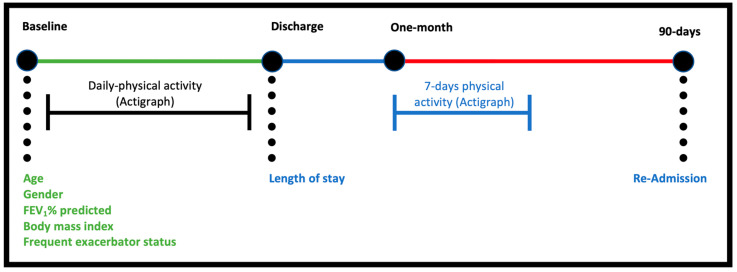
Data collection overview: black circles denote data collection points, coloured horizontal line depicts the inpatient (green), post-discharge (blue) and follow-up (red) study phases. Note: FEV_1_% predicted = percentage predicted of forced expiratory volume in 1 s.

**Figure 2:**
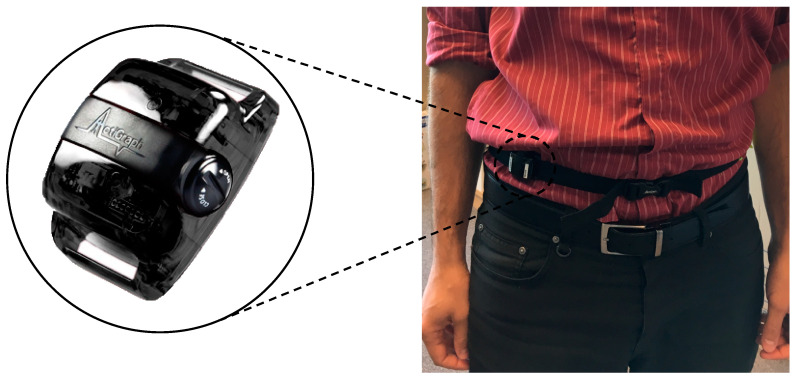
Actigraph wActiSleep-BT device (**left**) and location of wear (**right**).

Figure 1 summarises the data collection process. Demographic characteristics were collected following recruitment. Clinical outcomes (described further in Table 1) were collected from medical records at discharge, 1 month post-discharge, and 90 days post-discharge. Physical activity data collection occurred from recruitment until discharge via a wActiSleep-BT (GT3X model) (Actigraph, Pensacola, FL, USA) device attached to an elastic waist belt [21] worn on the dominant hip (shown in Figure 2) throughout the admission at all times unless removal was necessary (e.g., showering, imaging procedures, discomfort). After the one-month follow-up review, participants were also asked to wear the device in the same way for one week.

### 2.3. Data Management

All Actigraph data were downloaded using ActiLife TM software v6.13.3 (Actigraph, Pensacola, FL, USA), converted from raw form (units of gravity collected at 30 Hz) into 60-second epochs, and cleaned to remove irrelevant times (e.g., periods of non-wear and device movement occurring after removal from patient). A day was defined as occurring between 00:00 and 23:59, with all PA data collected during this time deemed eligible for inclusion as long as patients wore PA monitors for ≥8 h/day between the hours of 07:00 and 22:00 [17,22], validated using default Troiano (2007) algorithms [23]. Non-wear times were excluded from analysis. Inpatient data were not sleep-scored in ActiLife due to the challenges of detecting daytime sleep in participants anticipated to be highly sedentary in bed for long parts of the day and our decision to restrict analyses to a daytime hour range. Energy expenditure was calculated using the Sasaki and Freedson Adult VM3 Combination (2011) algorithm [24], and METs were defined according to the Freedson Adult (1998) algorithm [25]. Cut-off points used to define different intensities of PA were sedentary behaviour (<1.50 metabolic equivalent of task (METs)), 0–99 counts, 10 min minimum bout length; light PA (<3 METs), 0–2689 counts; moderate PA (3.00–5.99 METs), 2690–6166 counts; vigorous PA (6–8.99 METs), 6167–9642 counts; and very vigorous PA (>9 METs), ≥9643 counts. Day of the week (e.g., weekday/weekend) was not controlled for in analyses, as its relevance to PA accumulation in a hospital environment was deemed low (unlike in community-dwelling settings) [17]. At the one-month follow-up, a seven-day window of measurement was used in line with recommendations [17].

### 2.4. Analysis Plan

Stata SE v17.0 (StataCorp, College Station, TX, USA) was used for data analysis. Participant characteristics were described using descriptive statistics appropriate for the data type and distribution. Normality was inspected via histogram and Shapiro–Wilk tests. Alpha was set at 0.05 for all analyses.

Patterns of PA accumulation across different accelerometry metrics were summarised via descriptive statistics pertaining and represented graphically by summary box plots (step counts) and 100% stacked column graphs (percentage time spent in various PA intensities) as well as individualised line graphs. Trends across the first 7 hospital days were examined via one-way ANOVAs.

The clinical relevance of inpatient PA accumulation metrics was evaluated by examining their relationship with clinically relevant variables and subgroups divided on the basis of dichotomizing these variables. For this purpose, a combination of unpaired *t*-tests, Chi^2^ tests, and exploratory univariate regression or logistic regression analyses (depending on data type) were employed. Variables (and dichotomised subgroup thresholds) included (a) FEV_1_% predicted (GOLD stages 1–2 vs. 3–4), (b) LOS (≤/>5 days), (c) PA metrics at follow-up (and those who improved mean steps/day from discharge to one-month by a margin ≥1000—the minimum important difference threshold in stable COPD [22]), and (d) those who experienced a respiratory-related readmission or death within 90 days of discharge from hospital (censorship event). In line with the exploratory study aims, univariate regression analyses explored the relationship between eight clinical outcomes (dependent variables) and six ways that PA parameters (candidate predictors or independent variables) could be expressed during inpatient AECOPDs. These were (1) overall mean value across all available inpatient data (‘mean’), (2) PA data recorded from the first available day of capture during the inpatient period (‘first’), (3) PA data recorded from the last available day of capture during the inpatient period (‘last’), (4) lowest single-day value recorded during the inpatient period (‘nadir’), (5) maximum single-day value recorded during the inpatient period (‘maximum’), and (6) the difference between the last and first available day of data capture during the inpatient period (‘delta’). A summary of findings from the exploratory regression analyses is represented via a heat map, with shading corresponding to findings where *p* < 0.05. The proportion of variance (R^2^) explained by PA on the various outcome variables was noted for all regression analyses and is represented in Table S1 (Supplementary Materials).

## 3. Results

Sixty-eight recruited inpatient participants yielded at least one day of PA data for inclusion in the study, and fifty-one completed follow-up evaluations. Reasons for loss to follow-up were multifactorial (e.g., declined, not able to attend outpatient appointments, not responsive to contact methods). Participant demographics and inpatient management are summarised in Table 2 and Table 3.

### 3.1. Characterising PA Accumulation Patterns

**Figure 3:**
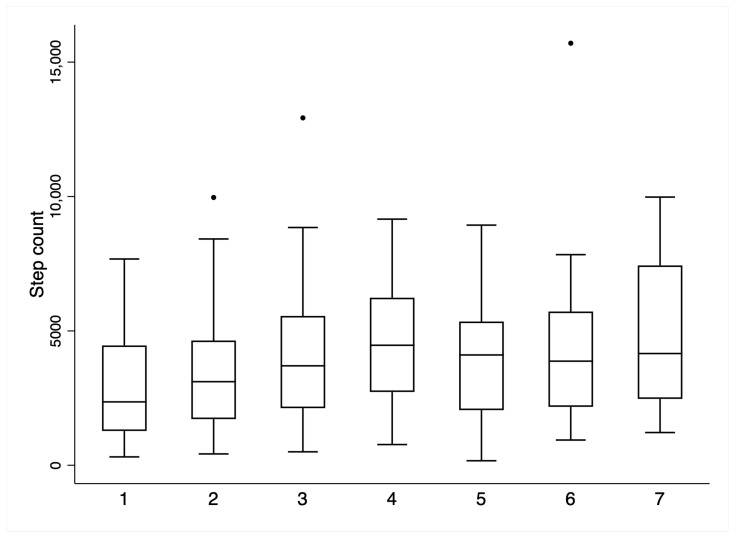
Inpatient step counts (*Y*-axis) per day (*X*-axis). Black circles denote outliers. Note: ANOVA *p* > 0.05.

**Figure 4:**
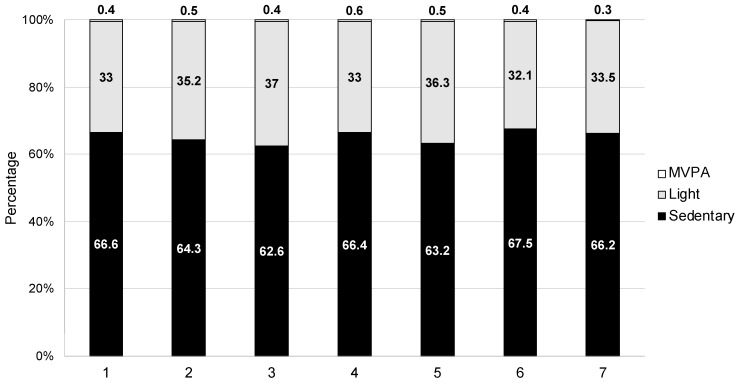
Proportion of time between 07:00 and 22:00 spent in different PA intensities per hospital day (*X*-axis). Note: Percentages calculated as a product of total device wear time and involving all available data (not just participants admitted for 7 days). All ANOVAs evaluate differences in time spent in various intensities over time *p* > 0.05. MVPA = moderate-to-vigorous physical activity.

**Figure 5:**
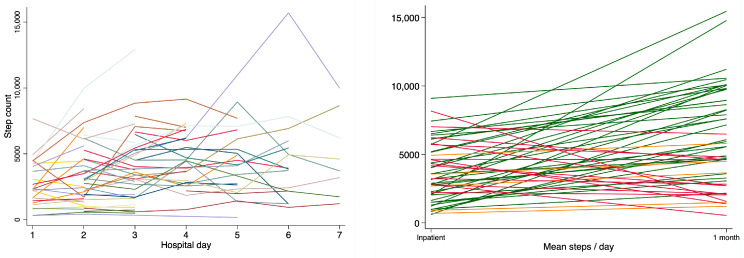
Individual PA trajectories: (**left**) daily step counts during the inpatient hospital admission (coloured lines denoting individual participants); (**right**) mean inpatient step count during inpatient admission and one-month follow-up (green lines denote improvement ≥ 1000 steps/day; orange lines denote improvement < 1000 steps/day; red lines denote decline in steps/day).

PA accumulation across both the inpatient and follow-up phases is summarised in Table 4. Improvements were observed across all PA metrics (*p* < 0.001 for all), with 33/51 (65%) participants achieving a gain in steps/day in excess of the minimally important different threshold for people with stable COPD (1000 steps/day [22]). No participant met international recommendations for PA [7] at any time during the study. The proportion of people who achieved this improvement threshold was numerically greater in those who had GOLD stages 1–2 (17/22, 77.2%) vs. 3–4 (13/25, 52%); however, this did not reach statistical significance (Chi^2^ *p* = 0.072). Patterns of PA accumulation for mean steps/day (Figure 3) and time spent in different-intensity PA (Figure 4) across individual days of the inpatient period demonstrated little day-to-day variability (all within-group ANOVA *p* > 0.05). Vast variability was observed, however, at the individual level (Figure 5).

### 3.2. Clinical Relevance of Inpatient PA Accumulation

Unpaired *t*-tests (Table 5) revealed that those with prolonged admission durations (>5 days) spent more time (minutes) sedentary per day in hospital (69.0 min (11.8); n = 26) and less time engaged in light-intensity PA (30.6 min (11.6)) compared to those with shorter admission durations (≤5 days) (59.9 min (12.4); n = 42; *p* = 0.004 between groups and 39.6 min (12.3); *p* = 0.004 between groups, respectively). Time (minutes) spent in MVPA was higher in those with mild COPD (0.6 [0.24 to 0.95]; n = 25) compared to those with severe COPD (0.3 [0.16 to 0.69]; n = 36; *p* = 0.016 between groups).

**Figure 6:**
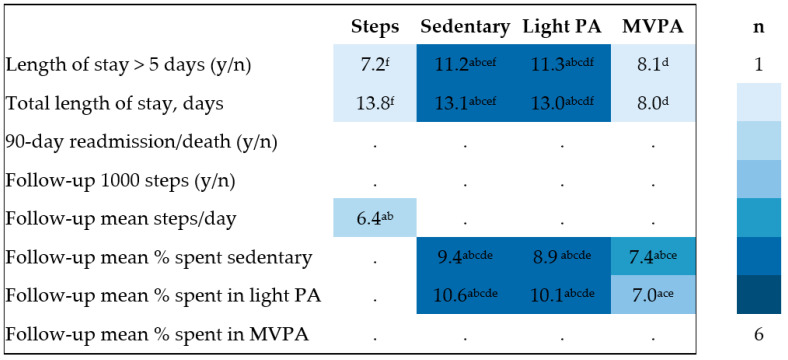
Summary of inpatient PA influence on follow-up outcomes (‘heat map’). Note: Colour shading reflects the number of statistically significant findings from regression analyses as per the figure legend. Superscript letter(s) indicate which form of PA parameter metric expression was statistically significant according to six possible variations: ^a^ ‘mean’, ^b^ ‘first’, ^c^ ‘last’, ^d^ ‘nadir’, ^e^ ‘maximum’, ^f^ ‘delta’; full details in Section 2.4. Data values reflect the arithmetic mean of the coefficient of determination (explanatory power) *R*^2^ and pseudo *R*^2^ values from statistically significant findings. Full details are provided in Table S1 (Supplementary Materials). MVPA = moderate-to-vigorous physical activity; PA = physical activity. Follow-up 1000 steps refers to the status (yes/no) of achieving an improvement >1000 mean steps/day from discharge to follow-up.

Exploratory regression analyses revealed that few PA metrics demonstrated meaningful relevance or statistically significant predictive ability for any clinical variables, with the exception of time spent in light PA (Figure 6). Exploratory power was low for all analyses across all outcomes (R^2^/pseudo R^2^ values ranging from 6% to 22%) with little difference apparent across the six different methods used to express each PA metric during the inpatient period (described further in Table S1 in the Supplementary Materials).

## 4. Discussion

This study offers a detailed evaluation of objective physical activity data for people hospitalised due to AECOPD. It highlights some of the first data supporting inpatient PA as a relevant factor impacting some clinical outcomes, but reveals potentially important differences in the relationship between different PA metrics and various clinical outcomes. This helps address known gaps in our knowledge related to inpatient PA [14] and supports a role for further enquiry to determine how we should best integrate such knowledge within future clinical care.

Our data reinforce prior observations that most people with AECOPD undertake concerningly low levels of PA during the period of hospitalisation [10,11,12,15]. The lack of change in PA, on average, across the duration of hospitalisation appears to lend support to recent recommendations by Orme et al. [11] that PA measurement on any single day of hospitalisation might provide adequate insight into typical PA over the course of admission for AECOPD. Our comparison between various PA metrics suggests this recommendation likely applies equally across the range of commonly measured PA metrics (e.g., steps, time spent in light/moderate PA, time spent sedentary) and the various ways each could be expressed (‘mean’, ‘first’, ‘last’, ‘nadir’, ‘peak’ or ‘delta’ values), where data are matched according to hospital day across a sample. The clinical and/or research applicability of this knowledge, however, may be contextually dependent. For example, where a crude cross-sectional evaluation of PA for a cohort of patients with AECOPD is acceptable, a single day of data collection may be justified. If more nuanced insights are desired at the individual patient level, our observations of large day-to-day inter- and intra-subject variability (Figure 5) suggest alternate and/or supplemental information may be indicated.

Minimal guidance exists to help determine which PA metric might be the most appropriate when evaluating PA during AECOPDs. Our univariate logistic regression analyses suggest that the way the metric is expressed (e.g., ‘mean’, ‘first’, ‘last’, ‘nadir’, ‘peak’, ‘delta’ value) confers a relatively low impact on analysis findings. Time spent in sedentary behaviour and light PA, however, was high, and these metrics demonstrated a greater number of associations with clinical variables than other PA metrics (Figure 6). These might therefore seem sensible outcome choices for studies seeking to explore the impact of PA on clinical outcomes. No PA parameter (e.g., steps, time spent sedentary or in light or MVPA) demonstrated superiority in terms of discriminatory potential over another (Table 4). This may infer that decisions regarding such issues could be left to the discretion of the individual clinician or researcher. The small amount of time observed to be spent in MVPA could suggest that it is a potentially suitable candidate target with a large scope for improvement in future PA intervention studies during AECOPD. While our data suggested a degree of spontaneous recovery of PA from discharge to one month later existed (Figure 5), future studies seeking to better understand and predict recovery of PA after AECOPDs are still essential. This is despite well-known challenges of conducting interventions during this phase [28,29].

Determining the best strategy to modify PA during AECOPD is a challenging and under-researched issue. Modifying time spent in light PA and/or breaking up bouts of prolonged sedentary behaviour seems attractive in terms of feasibility (i.e., ‘low hanging fruit’) but might necessitate greater dosages of sustained change in order to translate into health benefits. By contrast, increasing the duration of time spent in MVPA could be more difficult to achieve, but might elicit more potent effects on clinical outcomes in a shorter time (Table 4). This may have important implications for future interventions including rehabilitation targeting improvements in PA during AECOPDs. Few studies have directly targeted PA during AECOPD, with prior authors focusing on exercise training during AECOPD [30,31] breaking sedentary behaviour after AECOPD [29] or improving PA recovery after AECOPD [32]. Targeting MVPA during AECOPD may require careful consideration of appropriate training modalities (e.g., lower limb seated strengthening exercises) to minimise complications such as desaturation (which occurs frequently in walking). Bedside-based exercise can also prove challenging to detect via objective accelerometry, with devices needing to either distinguish between different body and joint positions (e.g., sitting vs. standing vs. cycling) and/or measure energy expenditure (e.g., via indirect calorimetry or galvanic skin response) if, for example, isometric training was to be performed. Many PA measurement devices offer differing features that can make one more suitable to a specific situation than another (particularly in the commercial market). It is therefore challenging to advocate the use of any one specific type or brand over another. Accelerometry is also not yet a widely accepted component of standard clinical care in most inpatient hospital settings. Simpler tools such as pedometers, commercial wearables (including technology embedded in smartphones and smartwatches), or even surveys may have a possible role to assist in this evaluation; however, more research is needed to validate such approaches in the context of AECOPDs. While survey methods of PA evaluation have known issues such as recall bias [33], it is plausible that the low levels of day-to-day PA variability at a sample level as well as the unique environmental and social constraints of a hospital setting could mean such limitations are less relevant for PA evaluation during AECOPD and might warrant further scrutiny. Efforts to redress low PA during AECOPD are likely, however, to require a multifaceted and comprehensive focus. Three key elements previously proposed as important to address to improve PA in hospitals include environmental context, resources, and social influences [13].

Several areas remain to be explored for future research. These include issues related to the precise patterns characterising PA accumulation during hospitalised AECOPDs (e.g., nature of PA bouts or ‘fragmentation’), examination of the drivers underpinning PA behaviours (e.g., is it motivated by activities of daily living or physiotherapy treatments?), how clinical care can optimise PA accumulation, and how hospital environments can be better designed to promote more active inpatient stays. Such insight might plausibly lead to clearer opportunities to engage with interventions seeking to improve outcomes for this patient group.

### Limitations

The present study represents an examination of an a priori hypothesis that sought to explore nuanced differences in the relationships between PA data and clinical outcomes in people admitted to hospital with AECOPD. Data collection and analysis were not planned to meet formal pre-specified sample sizes or power calculations. An example of how this limited findings was the inability to yield sufficient data relating to the proportion of participants who met international recommendations for daily PA [7] (which no one met at any time). Future studies are likely to be necessary in order to accurately determine the external validity and relevance of our findings for future clinical practice. The Actigraph device used in our study may also have failed to capture some potentially relevant PA data (e.g., bedside sit/stand exercise or time associated with showering). While this is a common limitation, alternatives such as the ActivePAL3 micro (PAL Technologies, Glasgow, Scotland, UK) [34] or SenseWear armband [11,15] can overcome such limitations and have been used during other AECOPD studies. Finally, the potential impact, both in magnitude and direction of effect, of attrition on data collection during daily inpatient PA measurement and follow-up evaluation is difficult to ascertain. Additional data from larger studies would assist in verifying our observations.

## 5. Conclusions

Physical activity can be accurately assessed via accelerometry during AECOPDs, but different PA metrics may confer differing levels of clinical relevance depending on the specific purpose of its evaluation. The present data suggest that no individual PA metric or method used to express PA during AECOPD appears more useful than another in terms of its relation to clinical outcomes. Further work is needed before routine measurement of PA during AECOPD is established in clinical practice.

## Figures and Tables

**Table 1:** Types of data collected for analysis.

Type of Data	Units of Measurement/Analysis Characteristics
Exposure variable: Physical activity	
Physical activity amount/duration	Steps/time spent active per day
Sedentary behaviour	Time spent sedentary per day
Physical activity intensity	MET-derived cut-offs (light, moderate, vigorous)
Outcome variables: Clinical outcomes	
Length of stay	Days
90-day readmissions	Yes or no
PA at one-month follow-up	Steps/day
Covariates	
Age	Years, days
Body mass index	Kg/m^2^
Gender	Male, female, non-binary
Frequent exacerbator status	0 or ≥1 prior event in the last 12 months
FEV_1_% predicted	FEV_1_% predicted

Note: MET = metabolic equivalent of task; FEV_1_% predicted = percentage predicted of forced expiratory volume in 1 s.

**Table 2:** Participant demographic characteristics.

Descriptive Variable	N = 68 (at Recruitment)
Age (years)	69.2 (9.0)
Gender:	
Male	38 (56%)
Female	30 (44%)
Body mass index (kg/m^2^)	27.2 (7.5)
FEV_1_% predicted	45.0 (17.7)
TLCO % predicted	44.2 (20.3)
Smoking status:	
Current	17 (25%)
Former	49 (72%)
Never	1 (1.5%)
Pack/years	41.8 [25.1, 55.5]
GOLD Stage:	
I	3 (5%)
II	22 (35%)
III	24 (39%)
IV	13 (21%)
Hypertension	40 (59%)
Ischaemic heart disease	21 (31%)
Diabetes	8 (12%)
Anxiety	25 (37%)
Depression	22 (32%)
Usual respiratory care:	
LABA	63 (93%)
LAMA	63 (93%)
ICS	57 (84%)
Long-term oxygen therapy	15 (22%)
Dyspnoea (eMRC Dyspnoea Scale):	
2	17 (25%)
3	18 (26%)
4	17 (25%)
5a	11 (16%)
5b	4 (6%)
No. exacerbations past year	1.0 [1.0, 3.0]
Frequent exacerbator status (2+ in last year) (n, %)	19 (32%)

Data are n (%) or median [inter-quartile range]. eMRC Dyspnoea Scale = Extended Medical Research Council Dyspnoea Scale [26]; FEV_1_% predicted = percentage predicted of forced expiratory volume in 1 s; GOLD = Global Initiative for Chronic Obstructive Lung Disease classification; ICS = inhaled corticosteroid; LABA = long-acting beta_2_-agonist; LAMA = long-acting muscarinic antagonist; TLCO = transfer factor of carbon monoxide. Data are mean (SD) or median [IQR]; variable percentages that total <100% reflect missing data.

**Table 3:** Overview of inpatient management.

Descriptive Variable	N = 68 (at Recruitment)
Exacerbation type *:	
1	20 (29%)
2	16 (24%)
3	26 (38%)
Inpatient corticosteroid use (IV/oral)	59 (87%)
Inpatient antibiotic use (IV/oral)	60 (88%)
Evidence of chest x-ray consolidation	18 (26%)
Use of BiPAP in emergency department	19 (28%)
Use of BiPAP on ward	16 (24%)
Length of stay (days)	4.0 [3.0, 7.0]

* Exacerbation type defined according to Anthonisen criteria [27]. BiPAP = bi-level positive airways pressure (non-invasive ventilation); Data are mean (SD) or median [IQR]; variable percentages that total <100% reflect missing data.

**Table 4:** Daily inpatient and follow-up PA accumulation.

	Day 1 (N = 31)	Day 2 (N = 47)	Day 3 (N = 40)	Day 4 (N = 26)	Day 5 (N = 21)	Day 6 (N = 16)	Day 7 (N = 8)	Inpatient Total (N = 68)	Follow-Up Total (N = 51)
Discharge day, n	-	7	19	11	5	7	7	-	-
Step count	2995.5 (2040.2)	3476.0 (2240.0)	4119.1 (2568.9)	4542.7 (2121.8)	4012.0 (2387.1)	4587.6 (3623.0)	4915.9 (3154.1)	3817.0 (2144.2)	6173.7 (3564.6)
Mean % sedentary time	66.6 (12.6)	64.3 (14.0)	62.6 (15.6)	66.4 (11.8)	63.2 (16.5)	67.5 (17.7)	66.2 (22.6)	63.4 (12.9)	49.4 (14.7)
Mean % light time	33.0 (12.3)	35.2 (14.0)	37.0 (15.4)	33.0 (11.7)	36.3 (16.2)	32.1 (17.5)	33.5 (22.5)	36.2 (12.7)	49.3 (14.0)
Mean % MVPA time	0.4 (0.6)	0.5 (0.7)	0.4 (0.6)	0.6 (0.8)	0.5 (0.5)	0.4 (0.3)	0.3 (0.3)	0.5 (0.6)	1.3 (1.8)

Note: Data are mean (SD). ANOVAs performed between day 1 and day 7 all *p* > 0.5. Paired *t*-tests comparing follow-up to inpatient data on available participants *p* < 0.001 for all. MVPA = moderate-to-vigorous physical activity.

**Table 5:** Follow-up metrics.

Factor	N	Mean Steps/Day	*p*-Value	Mean % Time in Sedentary	*p*-Value	Mean % Time in Light	*p*-Value	Mean % Time in MVPA	*p*-Value
Censor * no	48	4175.7 (1996.0)	0.15	62.6 (13.7)	0.75	36.8 (13.5)	0.80	0.3 [0.2, 0.8]	0.403
Censor * yes	17	3322.4 (2289.3)		63.8 (10.8)		35.9 (10.9)		0.3 [0.2, 0.5]	
LOS < 5	42	3892.5 (2210.0)	0.71	59.9 (12.4)	0.004	39.6 (12.3)	0.004	0.3 [0.2, 0.7]	0.387
LOS ≥ 5	26	3695.0 (2070.4)		69.0 (11.8)		30.6 (11.6)		0.3 [0.2, 0.6]	
GOLD 1 and 2	25	3712.0 (2040.2)	0.47	62.2 (13.2)	0.78	37.1 (12.9)	0.84	0.6 [0.2, 0.9]	0.016
GOLD 3 and 4	36	4121.2 (2254.6)		63.2 (13.5)		36.5 (13.5)		0.2 [0.2, 0.4]	

Note: Mean % time MVPA for GOLD 1 and 2 and GOLD 3 and 4 is median [IQR]. All other values are mean (SD). * Censor event defined as any respiratory-related readmission or death within 90 days of discharge from hospital. GOLD = Global Initiative for Chronic Obstructive Lung Disease criteria; LOS = length of hospital stay; MVPA = moderate-to-vigorous physical activity.

## Data Availability

The data in this study are not publicly available as it was not communicated to participants during the consent process or approved by the human research ethics committee.

## Supplementary Materials

The following supporting information can be downloaded at https://www.mdpi.com/article/10.3390/jcm12154914/s1: Table S1: Results from exploratory linear/logistic regression analyses evaluating the ability of inpatient PA variables to predict clinical outcomes when expressed in various ways.
