# Supplementary material for: Physical Activity Profiles among Patients Admitted with Acute Exacerbations of Chronic Obstructive Pulmonary Disease
Source: J Clin Med. 2023 Jul 26;12(15):4914. doi: 10.3390/jcm12154914 (PMC10419862; doi:10.3390/jcm12154914)
Supplement: Supplementary file 1 [file jcm-12-04914-s001.zip › jcm-2467658-supplementary.pdf]

Supplement Table S1. Results from exploratory linear/logistic regression analyses evaluating the ability of inpatient PA variables to predict clinical outcomes when expressed in various ways.

Note: Each outcome depicted sequentially a-h. Data represents coefficient of determination explanatory power values (R^2 or Pseudo R^2).

Outcome a: Length of stay >5 days (y/n)

	Steps/day	Sedentary, % time/day	Light, % time/day	MVPA, % time/day
Overall mean	.	9.8	9.6	.
First available day	.	12	12.1	.
Last available day	.	7	6.8	.
Nadir inpatient value	.	.	18.7	8.1
Peak inpatient value	.	18.3	.	.
Delta (last - first data)	7.2	9.1	9.2	.
Mean	*7.2*	*11.2*	*11.3*	*8.1*

Outcome b: Length of stay, days

	Steps/day	Sedentary, % time/day	Light, % time/day	MVPA, % time/day
Overall mean	.	10.6	10.3	.
First available day	.	12.6	11.3	.
Last available day	.	10.9	10.6	.
Nadir inpatient value	.	.	22.0	8.0
Peak inpatient value	.	20.7	.	.
Delta (last - first data)	13.8	10.7	10.7	.
Mean	*13.8*	*13.1*	*13.0*	*8.0*

Outcome c: Censored for readmission or death within 90 days of discharge (y/n)

	Steps/day	Sedentary, % time/day	Light, % time/day	MVPA, % time/day
Overall mean	.	.	.	.
First available day	.	.	.	.
Last available day	.	.	.	.
Nadir inpatient value	.	.	.	.
Peak inpatient value	.	.	.	.
Delta (last - first data)	.	.	.	.
Mean	.	.	.	.

Outcome d: Mean >1000 steps/day change from discharge to follow-up (y/n)

	Steps/day	Sedentary, % time/day	Light, % time/day	MVPA, % time/day
Overall mean	.	.	.	.
First available day	.	.	.	.
Last available day	.	.	.	.
Nadir inpatient value	.	.	.	.
Peak inpatient value	.	.	.	.
Delta (last - first data)	.	.	.	.
Mean	.	.	.	.

Outcome e: Mean steps/day at follow-up

	Steps/day	Sedentary, % time/day	Light, % time/day	MVPA, % time/day
Overall mean	6	.	.	.
First available day	6.7	.	.	.
Last available day	.	.	.	.
Nadir inpatient value	.	.	.	.
Peak inpatient value	.	.	.	.
Delta (last - first data)	.	.	.	.
Mean	*6.4*	.	.	.

Outcome f: Mean % time spent sedentary/day at follow-up

	Steps/day	Sedentary, % time/day	Light, % time/day	MVPA, % time/day
Overall mean	.	11	10.6	7.5
First available day	.	9.5	8.8	6.3
Last available day	.	8.7	8.2	7.1
Nadir inpatient value	.	8.4	8.9	.
Peak inpatient value	.	9.3	8	8.6
Delta (last - first data)	.	.	.	.
Mean	.	*9.4*	*8.9*	*7.4*

Outcome g: Mean % time spent in light PA/day at follow-up

	Steps/day	Sedentary, % time/day	Light, % time/day	MVPA, % time/day
Overall mean	.	12.6	12.2	6.8
First available day	.	10.4	9.8	.
Last available day	.	10	9.6	6.4
Nadir inpatient value	.	10.2	9.3	.
Peak inpatient value	.	9.6	9.8	7.8
Delta (last - first data)	.	.	.	.
Mean	.	*10.6*	*10.1*	*7.0*

Outcome h: Mean % time spent in MVPA/day at follow-up

	Steps/day	Sedentary, % time/day	Light, % time/day	MVPA, % time/day
Overall mean	.	.	.	.
First available day	.	.	.	.
Last available day	.	.	.	.
Nadir inpatient value	.	.	.	.
Peak inpatient value	.	.	.	.
Delta (last - first data)	.	.	.	.
Mean	.	.	.	.
